# Pathological processing of sentinel lymph nodes in endometrial carcinoma — routine aspects of grossing, ultra-staging, and surgico-pathological parameters in a series of 833 lymph nodes

**DOI:** 10.1007/s00428-022-03377-6

**Published:** 2022-07-19

**Authors:** Tilman T. Rau, Mona V. Deppeler, Lucine Christe, Franziska Siegenthaler, Sara Imboden, Andrea Papadia, Michael D. Mueller

**Affiliations:** 1 Institute of Pathology, University Hospital Düsseldorf, Moorenstr. 5, 40235 Düsseldorf, Germany; 2 Institute of Pathology, University Bern, Murtenstrasse 31, 3008 Bern, Switzerland; 3 Department of Gynecology and Obstetrics, Inselspital University Hospital and University, Bern, Switzerland; 4 Department of Gynecology and Obstetrics, Regional Hospital Lugano, Lugano, Switzerland

**Keywords:** Endometrial carcinoma, Sentinel lymph node, Frozen section, Ultra**-**staging

## Abstract

Sentinel lymph nodes are widely accepted in the treatment of endometrial carcinoma. Whereas surgical aspects are well studied, the pathological work-up in terms of grossing, frozen section, and the so-called ultra-staging is still a matter of debate. This results in conflicting national or center-based recommendations. In a series of consecutive 833 sentinel lymph nodes from 206 patients in endometrial carcinomas, we compared three different grossing techniques and the use of frozen section in terms of anatomy, detection rates, and survival. In total, 42 macro-metastases, 6 micro-metastases, and 25 nodes with isolated tumor cells were found. Lymph nodes affected at least with micro-metastasis were about 0.5cm enlarged. Detection rates in lamellation technique increased with a step of 5.9% to 8.3% in comparison to bi-valved or complete embedding. The lamellation technique presented with a slight beneficial prognosis in pN0 subgroup (OS, *p*=0.05), which besides size effects might be attributed to trimming loss. In frozen section, this effect was less pronounced than expected (OS, *p*=0.56). Ultra-staging only revealed additional micro-metastases and isolated tumor cells. Exclusively, macro-metastases showed poor survival (*p*<0.001). In multivariate analysis, T-stage, subtype, and lympho-vascular invasion status outperformed this staging parameter significantly. Grossing of sentinel lymph nodes is the most essential step with evidence to prefer lamellation in 2 mm steps. Step sectioning should consider widely spaced protocols to exclude macro-metastases. Frozen sections might add value to the intra-operative assessment of endometrial carcinoma in selected cases. The excellent biological behavior of cases with isolated tumor cells might question the routine application of pan-cytokeratin as ultra-staging method.

**Supplementary Information:**

The online version contains supplementary material available at 10.1007/s00428-022-03377-6.

## Introduction

Endometrial cancer (EC) is the most common gynecological cancer in developed countries. Assessment of regional lymph nodes is an essential part of staging EC to tailor adequate treatment options as around 10% of the patients show metastatic spread into the lymphatic system in contrast to otherwise low-risk tumor characteristics [30]. Historically, the additional surgical exploration of regional lymph nodes was either discarded or performed extensively with lymphadenectomy [6]. In between these extremes, the sentinel lymph node (SLN) concept serves as a compromise to achieve meaningful representative information about nodal status and to avoid the unnecessary burden of concomitant peri- and postoperative complications [6, 29, 36]. SLN mapping successfully reduces lymphedema and paresthesia, but shows similar oncological outcomes as complete lymphadenectomy due to low false negative rates [9, 25].

The SLN in EC has meanwhile gained substantial interest via the work of prominent cancer centers and prospective trials and is close to being accepted as a standard of care [12, 16, 22, 30, 39]. For instance, the National Comprehensive Cancer Network (NCCN) identified SLN mapping as considerable approach to early stage EC, which is defined by disease confined to the uterus and without any metastases detectible by imaging processes [5], which is paralleled by further national guidelines [13, 17].

However, the pathological processing varies firstly in macroscopic evaluation in the number, depth, and interval between gross slices, secondly by FFPE step sectioning with routine H&E staining with variable distances between 50 and 250 μm, and thirdly in the use of immunohistochemistry (IHC) to identify malignant cells not identified by H&E alone [10, 13, 22]. The most prominent protocols in use are from MD Anderson Cancer Center [16], Memorial Sloan-Kettering Cancer Center [30], or the Mount Sinai Health System [4] beyond others reviewed in more detail by Burg et al. [10].

The first attempts to achieve consensus are pronounced [22, 33], with mainly experience-based considerations, but with little support of pathology-driven experimental data comparing the different methods as follows. Two different ultra-staging protocols showed that one versus five levels of 250 μm with immunohistochemistry aside was not inferior in metastasis detection rates [15]. Additionally, meta-analysis found evidence for the better performance of perpendicular gross sectioning than longitudinal sectioning in the comparison of detection rates between studies [10].

**Fig. 1:**
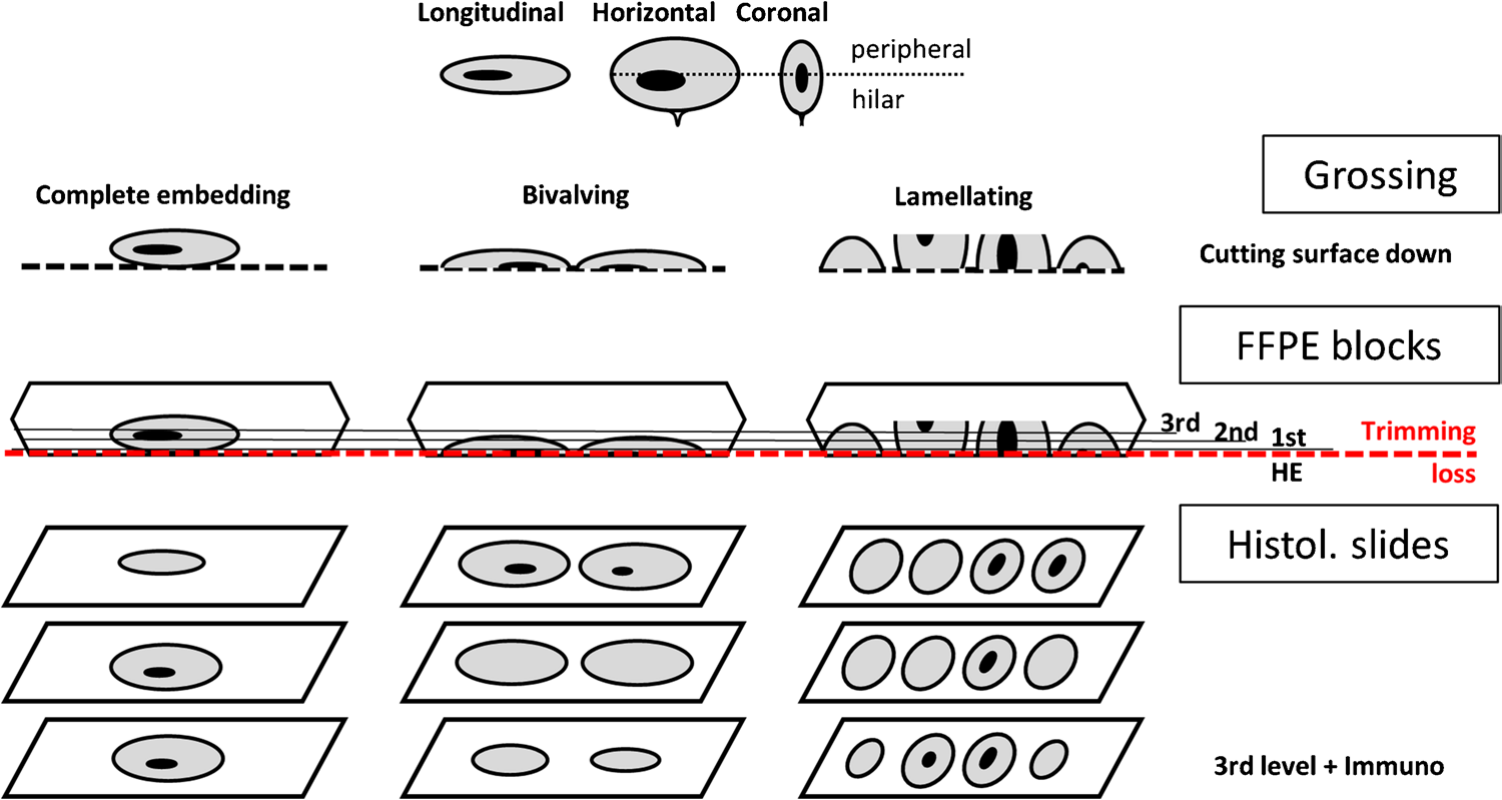
Concept of the three different grossing techniques and a possible impact on metastasis localization during histopathological processing. Adapted from Weaver et al. [7]. Note the distinction of peripheral versus hilar areas in a lymph node — used to describe anatomical differences in the presence of macro- and micro-metastasis, and isolated tumor cells

The SLN concept paper of Weaver et al. derived from the NSABP Protocol B-32 in breast cancer represents a well-known reference [42]. It starts logically with how to detect gross lymph nodes and what to expect from more intensified levels during ultra-staging. It was amended by us conceptually in only one assumption (Fig. 1, study concept). The most unlikely shape of a lymph node metastasis is a perfectly spherical object. Metastases before extracapsular extension follow the regular form of the lymph node, starting at the peripheral sinus, and are therefore stretched and elliptical in nature.

Our study reaches back to 2012, with few regulatory notes for pathologists on how to process SLN despite the minimum recommendations of 3 mm macroscopic slices, three levels of 200 μm, and immunohistochemistry.

With our study, we aim to (1) compare the grossing techniques of bread-loaf sectioning, longitudinal bi-valving, and complete embedding in terms of detection rates; (2) show the added value from first HE section to further steps; and (3) show the intra-operative SLN assessment in frozen section in terms of safety and precision.

## Material and methods

### Patient cohort

Patients diagnosed with EC between 2012 and 2021 that had undergone nodal staging with sentinel node mapping, with or without lymphadenectomy, at the University Hospital in Berne, Switzerland, were included.

### Data monitoring and definition of additional histopathological parameter

The corresponding primary tumors were re-assessed by TTR and LC. Histological subtypes, staging, and grades of tumors were evaluated according to the current WHO (2020) and FIGO (2017) criteria [2, 34]. Microcystic, elongated, and fragmented pattern (MELF) was assessed as putative prognostic parameter. Regarding the ESGO/ESMO risk classification, we distinguished between focal and extensive lymphatic invasion. Distinction from vascular invasion was based on HE features like presence of erythrocytes in vascular spaces or orphan artery signs. All patient characteristics can be taken from Table 1.

**Table 1:** Patient characteristics

	*n*	%
Histological subtype		
Endometrioid adenocarcinoma	174	84.5%
Serous carcinoma	13	6.3%
Mixed type carcinoma	5	2.4%
Carcinosarcoma	3	1.5%
Clear cell carcinoma	9	4.4%
Dedifferentiated carcinoma	2	1.0%
Tumor grading according to FIGO 2017		
G1	81	39.3
G2	63	30.6
G3	62	30.1
Tumor grading according to WHO 2019		
Low grade	144	69.9
High grade	62	30.1
T-stage		
T1a	128	62.1%
T1b	45	21.8%
T2	9	4.4%
T3a	17	8.3%
T3b	7	3.4%
N-stage		
N0	161	78.2 %
N0 i+	19	9.2 %
N1 mi	3	1.5 %
N1	14	6.8 %
N2	9	4.4 %
M-stage		
M0	203	98.5 %
M1	3	1.5 %
Lymphatic invasion		
L0	166	80.6%
L1 focal	13	6.3%
L1 extensive	27	13.1%
Vascular invasion		
V0	193	93.7 %
V1	13	6.3 %
MELF pattern		
Present	50	24.3%
Absent	156	75.7%
Perineural invasion		
Pn0	204	99.0 %
Pn1	2	1.0 %
Residual status		
Rx	2	1.0 %
R0	204	99.0 %
R1	0	0 %
Tracer diffusion		
Focused SLN number per site	186	90.3%
Extended SLN number per site	20	9.7%
Indication		
SLN mapping for limited staging	97	47.1%
SLN combined with lymphadenectomy	109	52.9%
ESGO risk groups		
Low	92	44.7%
Intermediate	45	21.8%
Intermediate-high	33	16.0%
High	33	16.0%
Advanced	3	1.5%
Total	206	100 %

### Pathological gross processing

The grossing method, metrics of lymph nodes, the number of nodes per block, the counts of macroscopic slices, and the number of step sections were controlled retrospectively. This allows for a three-tiered analysis of SLN as completely embedded, longitudinal bi-valved, and bread-loaf perpendicular sectioned.

### Immunohistochemistry

Immunohistochemistry followed routine protocols for FFPE material. Here, 3 μm slides were stained with the following primary antibodies: pan-cytokeratin marker AE1/AE3 (1:400, M3515, Dako, Santa Clara, CA, USA) for ultra-staging.

### Pathological ultra-staging

An initially negative SLN was subsequently processed with ultra-staging, including a minimum of two further serial sections at a distance of 200 μm and immunohistochemical staining with pan-cytokeratin AE1/AE3. The definition of macro-metastases, micro-metastasis, and isolated tumor cells followed the recommendations of the AJCC. In brief, the thresholds in size are more than 2 mm for macro-metastasis, 0.2–2 mm for micro-metastasis, and less than 200 μm diameter for isolated tumor cells (i+). The terms low-volume disease and ultra-low volume disease were avoided as authors vary in their definitions [20, 21, 30, 38].

### Statistical analysis

Binomial distribution and confidence intervals were used for the comparison of detection rates. Proportional distributions were analyzed with the chi-square test. Unmatched samples were compared using the Student *t*-test. All *p* values are two sided and the statistical significance level was set at *p*<0.05. The clinical follow-up is described by overall survival (OS). The clinical outcome was evaluated in relation to age, histological type, tumor stage, nodal status, presence of lymph vascular invasion, occurrence, and type of nodal metastases. Survival rates were analyzed using log-rank tests and the plotting of Kaplan-Meier curves. Multi-variate analysis was performed using Cox-regression analysis.

## Results

### Dependencies from anatomic-pathological parameters

**Fig. 2:**
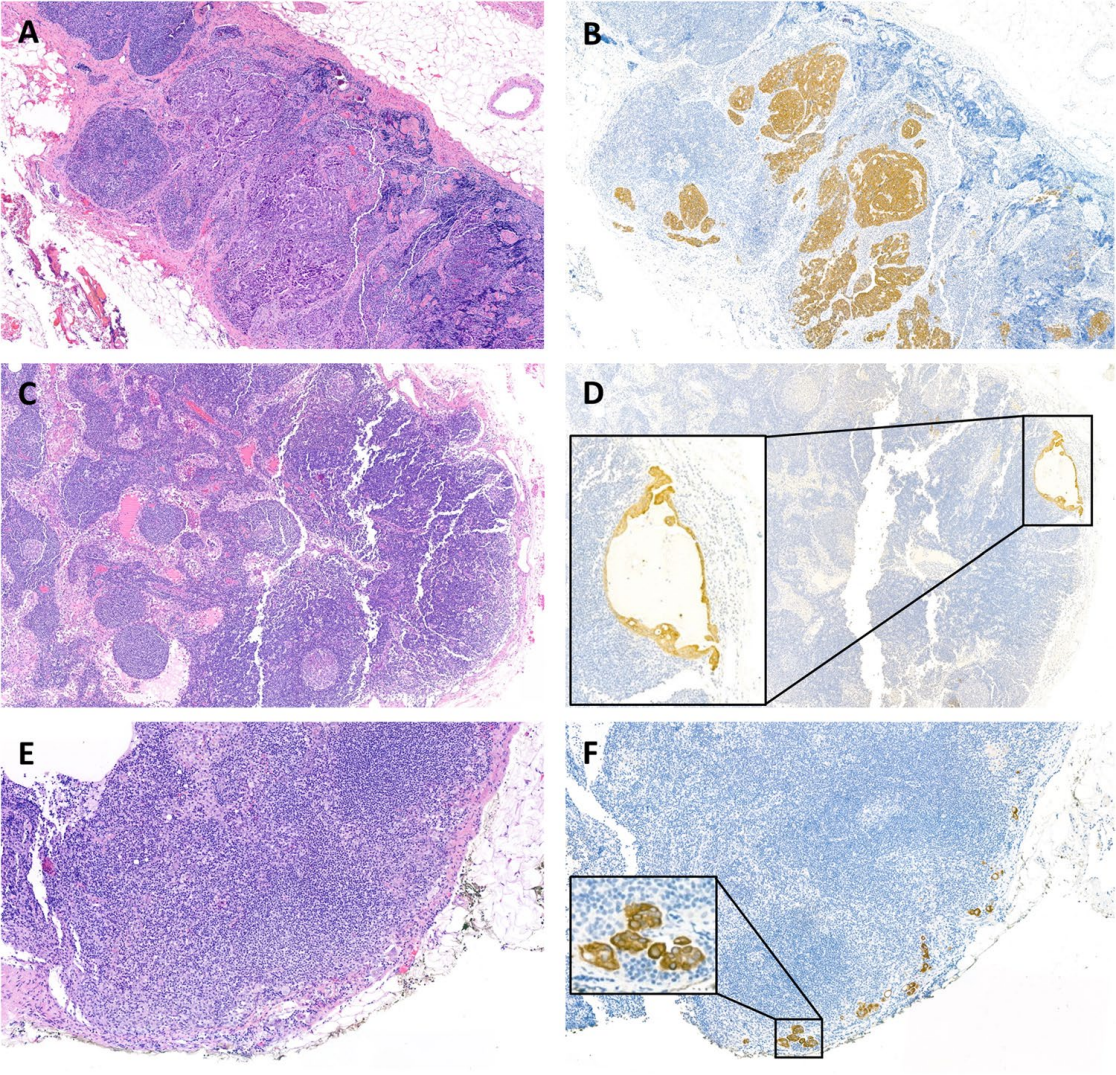
Examples of macro-metastasis (**A**, **B**); already visible in HE sections, newly detected micro-metastasis during ultra-staging (**C**, **D**); note the cytological and architectural atypia, and isolated tumor cells (**E**, **F**), respectively. Stained with conventional HE (**A**, **C**, **E**) and pan-cytokeratin (**B**, **D**, **F**)

In total, 833 SLNs were analyzed. Seventy-three metastatic SLNs were found, consisting of 42 macro-metastases, 6 micro-metastases, and 25 nodes with isolated tumor cells (Fig. 2). The explored anatomical regions are outlined in descending frequencies in Table 2. Several regions per patient were investigated with the SLN technique. Neither in surgical preparation, nor in positive rates side-specific differences could be found.

**Table 2:** Localization and side distribution of the 833 SLN

	Right				Left				Not noted				Total			
	pos.	%	Count	%	pos.	%	Count	%	pos.	%	Count	%	pos.	%	Count	%
Obturator fossa	26	9.6%	272	56.1%	17	8.5%	200	41.4%	0	0.0%	13	2.7%	43	8.8%	485	58.2%
External iliac artery	11	11.1%	99	47.6%	10	9.2%	109	52.4%			0	0.0%	21	10.1%	208	25.0%
Pre-sacral	3	10.3%	29	45.3%	0	0.0%	5	7.8%	0	0.0%	30	46.9%	3	4.7%	64	7.7%
Common iliac artery	1	3.4%	29	76.3%	1	11.1%	9	23.7%			0	0.0%	2	5.3%	38	4.6%
Para-aortic	0	0.0%	7	26.9%	1	14.3%	7	26.9%	2	16.7%	12	46.2%	3	11.5%	26	3.1%
Parametrial	1	33.3%	3	33.3%	0	0.0%	5	55.6%	0	0.0%	1	11.1%	1	11.1%	9	1.1%
Not oth. spec.			0	0.0%	0	0.0%	1	33.3%	0	0.0%	2	66.7%	0	0.0%	3	0.4%
Total	42	9.6%	439	52.7%	29	8.6%	336	40.3%	2	3.4%	58	7.0%	73	8.8%	833	100.0%

**Fig. 3:**
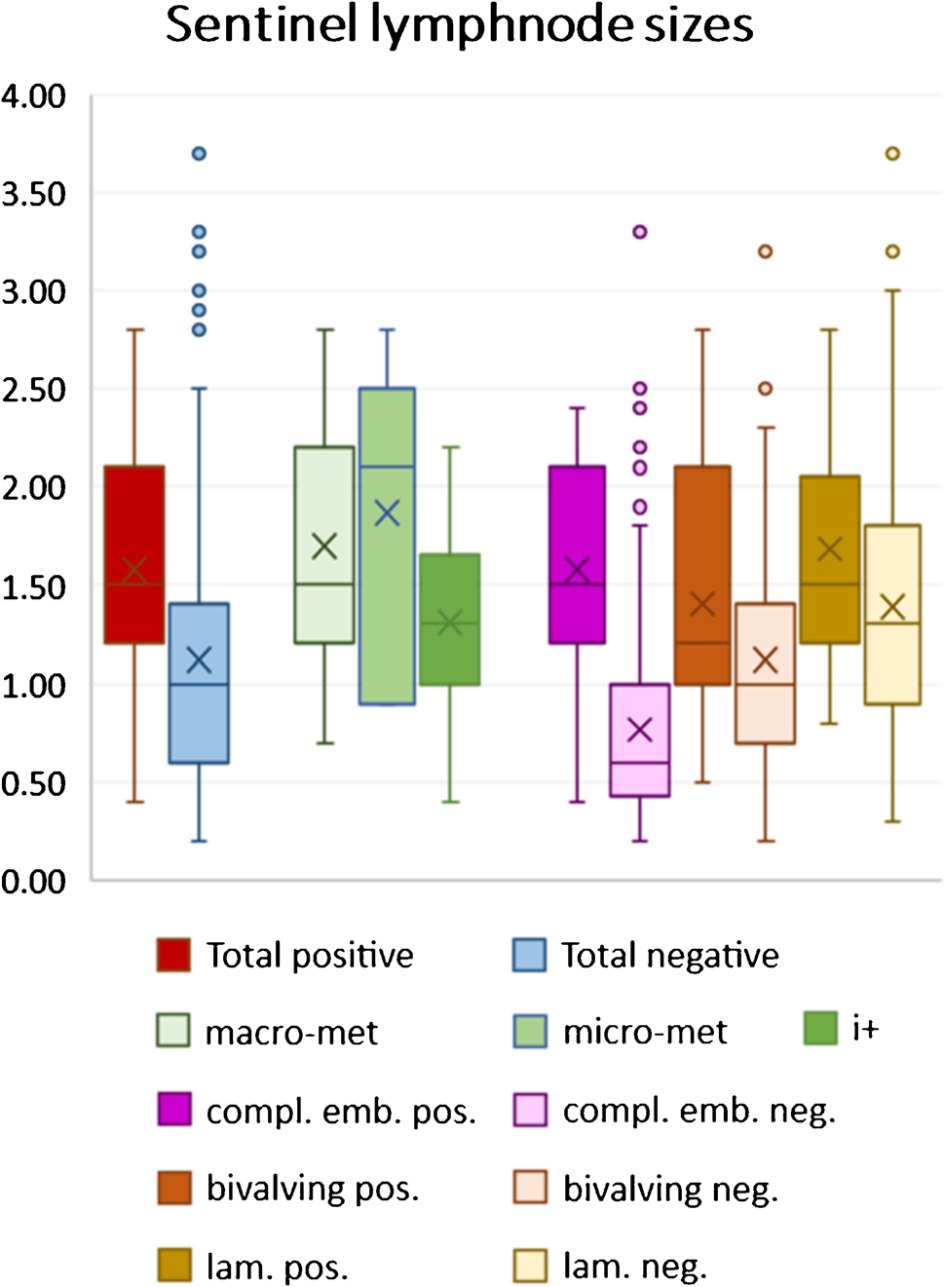
Boxplots of size differences between negative and positive lymph nodes — depicted in total, type of metastasis, and differences in pathological grossing technique

Of interest, metastatic lymph nodes were approximately 5 mm bigger in size than un-affected lymph nodes with means of 1.6 cm vs. 1.1 cm, respectively (*p*<0.001, Student’s *t*-test as followed). However, this accounted only for macro-metastasis (*p*<0.001) and micro-metastasis (*p*=0.008), but not for isolated tumor cells (*p*=0.17) (Fig. 3).

Following the lymphatic drainage within the lymph node (Fig. 1), we sited lymph node metastasis in the outer curvature and/or the central hilar position. The drift from outside to central areas in the lymph node increased from isolated tumor cells (82% to 18%) to micro-metastasis (63% to 37%) and further to macro-metastasis (51% to 49%), respectively.

### Effect of macroscopic preparation on metastasis detection rates

The grossing technique was separated into complete embedding (*n*=223, 26.8%), longitudinal bi-valving (*n*=314, 37.7%), or bread-loaf perpendicular sectioning to the lymph node axis (*n*=296, 35.5%). Of interest, the detection rate increased significantly from 4.9% (complete embedding) to 7.3% (bi-valving), reaching 13.2% (lamellation), respectively (*p*=0.002; chi-square). Compared to a ground truth of 13.5% average positive rates taken from a meta-analysis [10], this indicates a significant underperformance in the detection rates of complete embedding, as well as longitudinal bi-valving as a grossing technique (*p*<0.001, binomial test).

Increasing size of lymph nodes (Fig. 3) might contribute to a more intensified work-up, which can be best appreciated in the significant steps from negative completely embedded lymph nodes, to bi-valved and then lamellated lymph nodes with means of 0.8 cm, 1.1 cm, and 1.4 cm, respectively (each step *p*<0.001; Student’s *t*-test).

**Fig. 4:**
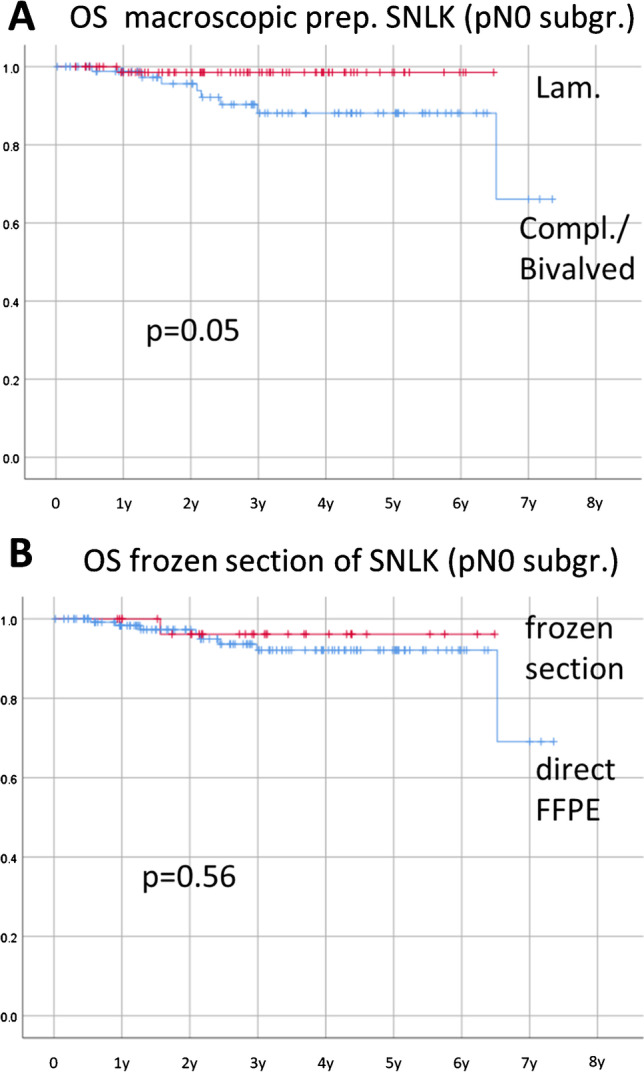
Kaplan-Meier curves of the nodal negative subgroup (pN0) as test for possible occult metastasis. **A** Focuses on grossing technique and **B** on frozen section application

As a surrogate test for possible occult metastasis, we performed a survival analysis of the pN0 subgroup in dependency from the grossing technique. Ninety-one cases with predominant work-up of the lymph nodes in complete or longitudinal bi-valving technique were contrasted with 115 cases using the preferred lamellation technique (Fig. 4). The less intensified macroscopic work-up of SLNs presented herein as a prognostic parameter and hence as a possible unfavorable parameter. In multivariate analysis, this difference was neither significant against the ESGO risk group (*p*=0.135, Cox regression), nor the single values of T-stage, lympho-vascular invasion (LVI) status, grading, and histological subtype. In our cohort, intensified macro-preparation was haphazardly less applied on non-endometrioid subtypes (chi-square, *p*=0.01), which might contribute to the survival effects and underpins the need for exact grossing techniques particularly in high-risk cases. No dependencies from other parameters could be shown (chi-square, *p*>0.05 in all combinations).

### Surgico-pathological effects on metastasis detection rates related to indication, tracer diffusion, and frozen section

The sentinel lymph node biopsy technique was either indicated as interim lymph node prior to lymphadenectomy or applied for limited nodal staging information (Table 1). Hence, the therapeutic role of SLN in terms of up-staging towards later lymphadenectomy cannot be answered with this study. Detection rates, reasons for indications, and grossing techniques showed no change over years.

The tracer used in this study was throughout indocyanine green. Tracer diffusion to more than five lymph nodes per site is a multi-factorial phenomenon, which might be linked to anatomical specialties, delayed injection to surgery times, and surgical training. Logically, the positivity rate was diluted as well from 10.1 to 3.9% on the lymph node level with trending significance (*p*=0.06; chi-square), but without identifying more positive cases (20.0% to 19.8%, *p*=0.98; chi-square). Of note, the grossing pathologist opted for less intensified grossing techniques in diffused tracer cases with more than 5 sentinel lymph nodes per site (4.7% instead of 19.5%; *p*=0.02; chi-square).

In total, 149 (17.9%) SLNs were assessed through frozen section analysis (Fig. 4), detecting 14 macro- and 2 micro-metastases. Two metastases were initially missed, but no false positive events occurred, leading to a sensitivity of 89% and specificity of 100%, with a positive predictive value of 100% and negative predictive value of 98.5%. The frozen section detection rate did not differ from the abovementioned ground truth of 13.5% (*p*=0.19, binomial distribution). Ultra-staging was not compromised, as all metastasis showed immune reactivity in pan-cytokeratin staining and acceptable HE images. Again, surrogate testing for missed occult metastasis was performed within the pN0 subgroup (Fig. 4). Survival in frozen section cases was not inferior to regular histological analysis.

### Effect of ultra-staging on metastasis detection rates

Those lymph nodes without metastatic involvement in the first HE section went into deepened ultra-staging procedures, resulting in 95.2% step-sectioned and 93.5% immunohistochemically stained SLNs.

The first HE in regular histology already detected 39 (92.9%) macro-metastases and 3 (50%) micro-metastases, which could be increased with step sectioning to 42 (100%) and 6 (100%), respectively. Pan-cytokeratin staining revealed isolated tumor cells in an additional 25 (100%) lymph nodes.

### Prognostic associations of macro-metastasis, micro-metastasis, and isolated tumor cells with T-stage, grading, LVSI, histological subtypes, and ESGO risk groups

Regarding each sentinel lymph node, an association between T-stage for risk of lymph node metastasis was given with stepwise increased rates of 4.9% for pT1a, 10.0% for pT1b, and 26.8% for ≥ pT2 (*p*<0.001; chi-square). Of interest, only the presence of macro-metastasis contributed to this association. Cases with isolated tumor cells did not show this distinction (*p*=0.79; chi-square) nor in combination with micro-metastasis (*p*=0.88; chi-square).

Regarding grading, the two systems of FIGO (2017) and WHO (2019) were analyzed. The three-tiered FIGO system was more informative for the prediction of lymph node metastasis with increased rates of 3.5% for G1, 12.5% for G2, and 11.2% for G3 (*p*<0.001; chi-square). In comparison, the increase from 7.7% low grade to 12.6% high grade in the WHO system was as significant but less pronounced (*p*<0.001; chi-square). Again, the association with grading was mainly based on macro-metastasis. Of note, isolated tumor cells were inversely associated with FIGO G2 (*p*=0.003; chi-square) and WHO low-grade cases (*p*<0.001; chi-square). Micro-metastasis could not be attributed to one of these two effects.

As expected, lympho-vascular invasion strongly predicted lymph node metastasis. A substantial increase from 4.2% without lymphangiosis to 6.9% in focal lymphangiosis to 32.8% for extensive lymphangiosis cases could be detected (*p*<0.001; chi-square) as well as an increase from 6.3% without vascular invasion to 29.9% in cases with vascular invasion (*p*<0.001; chi-square). Isolated tumor cells were over-represented in L0 and V0 cases (*p*=0.005, *p*=0.03; chi-square).

The microcystic elongated and fragmented pattern presented with an increase from 7.5 to 12.5% positivity rate (*p*=0.004; chi-square).

The distribution of metastatic events between endometrioid and non-endometrioid subtypes did not differ significantly (*p*=0.19, chi-square; Table 3). This accounts in detail for macro-metastasis, whereas isolated tumor cells and micro-metastasis were mainly found in the endometrioid subtype.

**Table 3:** Detected metastases in histological subtypes

Subtype	Macro	Micro	i+	Negative	Total	Ratio
Endometrioid	32	5	25	634	696	8.9%
Serous	5	0	0	66	71	7.6%
Mixed type	3	0	0	17	20	15.0%
Carcinosarcoma	0	0	0	10	10	0.0%
Clear cell	2	1	0	29	32	9.4%
Dedifferentiated	0	0	0	4	4	0.0%
Total	42	6	25	760	833	

Molecular data were not available within this study. Therefore, ESGO risk assessment was generally based on the combination of the abovementioned parameters. The significance of its predictive power for lymph node metastasis is highly significant (*p*<0.001, chi-square) and outlined in Table 4. Again, this finding is mainly based upon macroscopic metastasis with inverse association of isolated tumor cells to low risk cases (*p*=0.03; chi-square). The low number of micro-metastasis did not allow for a specific trend.

**Table 4:** Detected metastases in ESGO risk groups

	Macro	Micro	i+	Negative	Total
Low	0	2	11	371	384
Intermediate	3	0	7	152	162
Intermediate-high	0	1	1	101	103
High	36	3	5	124	168
Advanced-metastatic	3	0	1	12	16
Total	42	6	25	760	833

**Fig. 5:**
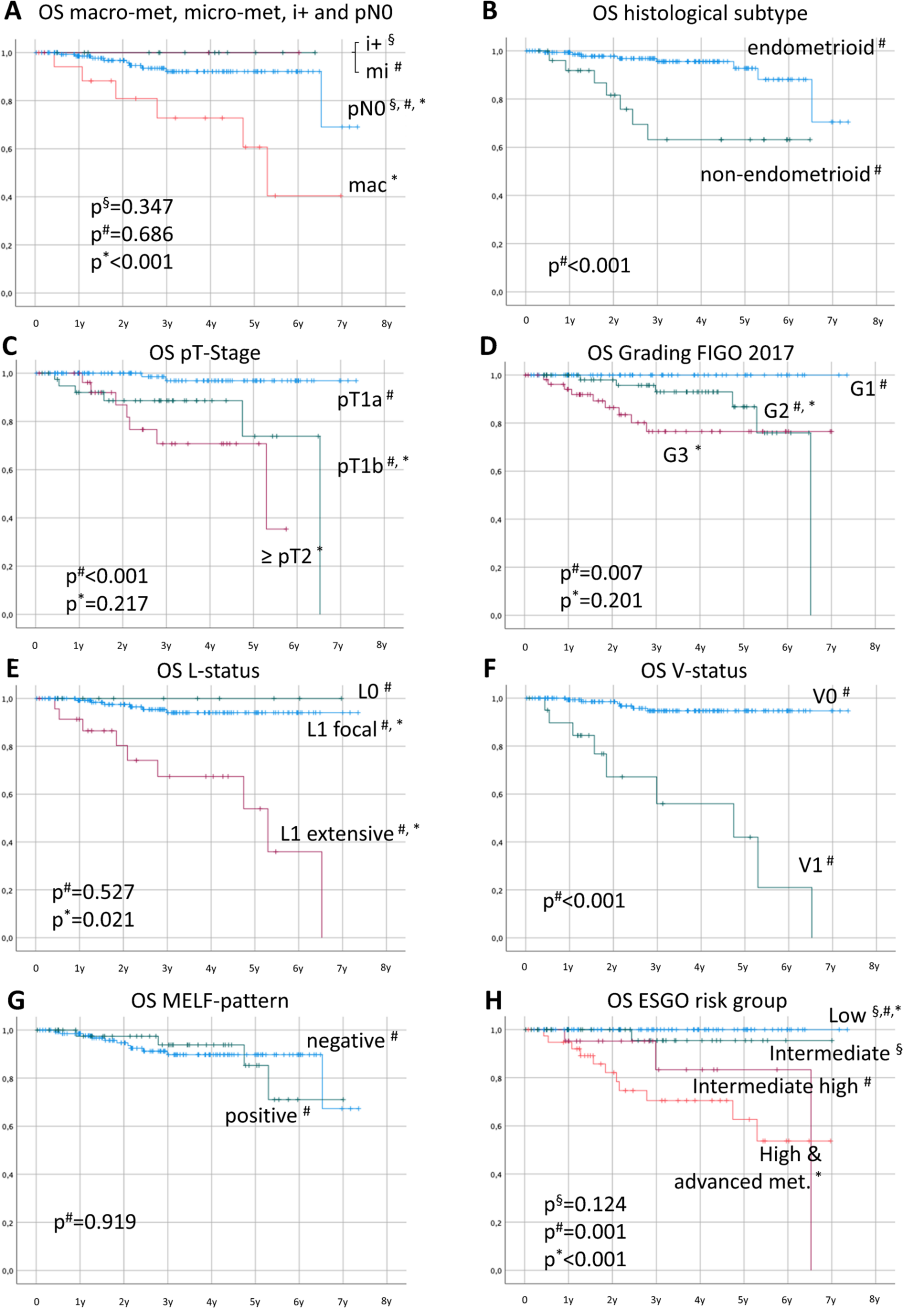
Survival curves of type of metastasis (**A**) in comparison to conventional risk parameters in EC, namely histological subtype (**B**), T-stage (**C**), grading (**D**), lympho-vascular invasion with differentiation into focal and extensive pattern (**E**), vascular invasion (**F**), MELF-pattern (**G**), and the combination made by the ESGO/ESMO risk classification — here without molecular data (**H**)

Furthermore, we analyzed data on the case level. Follow-up data were accessible for *n*=188 patients (mean 35.7 months, range 1–89). Survival analysis of T-Stage, grading, LVI, histological subtype, and ESGO risk group showed the expected discriminatory power. The detailed analysis of macro- and micro-metastasis, and isolated tumor cells showed an unfavorable prognosis for macro-metastasis only (Fig. 5).

In multivariate analysis with Cox regression T-stage (*p*=0.031, dichotomized at pT1a), LVI status (*p*=0.026) and subtype (*p*=0.002) remained the most influential independent variables. Grading contains inter-dependencies to the non-endometrioid subtype and was therefore not significant (*p*=0.933). However, neither additional parameters like MELF pattern, macroscopic work-up, or application of frozen sections, nor the combinations of macro- and micro-metastasis, or isolated tumor cells presented as an independent prognostic variable.

## Discussion

### Sentinel lymph node assessment in endometrial carcinoma — lessons learned from other entities

The sentinel lymph node concept is used to de-escalate clinical decisions in many tumor entities like breast carcinoma, melanoma, and Merkel cell carcinoma [3, 14, 19, 35]. Caution is necessary to transfer such knowledge to other entities like endometrial carcinoma. However, the three-dimensional logics of a SLN and its processing are universal and form a strong base of this manuscript [31, 32, 42, 43].

The most prominent surgical difference in endometrial carcinoma from other entities is the possibility of indocyanine green application instead of methylene blue or technetium labeling due to the subserosal anatomical sites in the intra-abdominal cavity and the fluorescence appearance in near infrared light during laparoscopic surgery [9, 27, 37].

### The role of frozen section in endometrial carcinoma — safe extensions towards intra-operative SLN evaluation

In the management of endometrial carcinoma, several pre-operative imaging approaches are currently in use. Intra-operatively, frozen section of the uterus can be applied to stratify for extensive lymphadenectomy [4, 36]. In these cases, the task for pathology is to assess depth of myometrial invasion, histological subtypes, and grading in a representative way [4]. Of note, pT3a situations with adnexal involvement are sometimes missed. Therefore, an outer inspection for tumor formations in the salpinx or ovary is mandatory before dissection of the uterus is performed and should encounter the differential diagnosis of two separate primary tumors [41]. The uncertainties of this intra-surgical assessment were recently outlined as 10% under- and 4% over-staged cases [4].

However, the combination of information available during surgery might help to improve clinical decision making. This includes direct processing of the SLN [1]. Some societies argue against this approach, because of the assumed loss of tissue and assumed technical difficulties of the later ultra-staging, namely immunohistochemistry [33]. This leads to less detailed scientific reports about a possible added value by frozen section, although some centers mention having included SLN after frozen section in their series as well [4, 28].

In our cohort, frozen section neither lowered the detection rates, nor impaired the consecutive ultra-staging or prognosis in the pN0 subgroup. Trimming loss is often attributed to frozen section, but occurs in FFPE blocks as well. Lamellation might serve as preventive measurement for both scenarios. Taking this into account, we regard intra-operative SLN processing as an optional procedure in EC.

### The priority of macro-staging of SLNs before ultra-staging

The macroscopic grid is the most important basic consideration in SLN work-up. As the AJCC defines 2 mm as the threshold of macro-metastasis, the thickness of macroscopic slices should logically cohere. Still, 3 mm lames are widely recommended and also applied by us, which seems to be a technical number rather following the depth of the FFPE mold [4, 23]. Of note, the lamellation technique decreased the *z*-axis of the slices significantly and could be easily lowered to 2 mm steps. However, advantages of the longitudinal sectioning used in several studies exist. It is more convenient as it is a quick grossing method with good grip of the specimen and less slicing. In real life, it will result in bi-valving of the majority of SLN as the smallest diameter in the longitudinal plane of the lymph node will regularly be less than 6 mm. Burg et al. compared the results of 11 studies with longitudinal sectioning versus 4 studies with bread-loaf perpendicular slicing and described higher detection rates as well [10]. This could even result in possibly missed occult metastasis with an influence on survival data, as in our cohort.

However, some cofounders for this effect could be identified. Firstly, a smaller size of the lymph node directs to less intensified grossing techniques, but could as well be an indicator for surgical sampling errors. Secondly, expected workload for the pathologist influenced the grossing technique. Reduced grossing procedures were found in cases with extensive tracer diffusion and in non-endometrioid cases, possibly as knowledge of a consecutive lymphadenectomy might have passed to the pathologist.

Despite these process-dependent reflections, there is a tumor biological argument of localized metastasis with increased size towards the central more hilar sites within the SLN, which makes them presumably more susceptible to the inevitable FFPE trimming loss from both mirror-like halves.

### Critical aspects about ultra-staging involvement in endometrial carcinoma

Micro-metastasis can be evident on every slide in the *x*- and *y*-axis. Of note, the first HE section has already an extremely high precision in metastatic detection. However, given a 2 mm sliced SLN, the following histological step sections will be a dichotomized test for micro-metastasis according to the chosen distances in the *z*-axis and the expected left-over material in the block [4]. Wide-spaced protocols with 200 μm distance might better ensure the maximum of 2mm thickness in the rest to avoid undetected macro-metastasis as a priority. In a recent meta-analysis, pure micro-metastasis with its 2.5% (61 of 2445) of cases seems to be rare in contrast to the presence of isolated tumor cells in 4.0% (99 of 2445), excluding the study of Ignatov et al. from the summary, which was purposely enriched in micro-metastases [24]. Micro-metastases already enlarge the affected lymph node, grouping them as macro-metastases rather than isolated tumor cells. Statistically, the 6 cases with micro-metastasis underpower our study to clarify the prognostic role of micro-metastasis. However, in the abovementioned multi-centric analysis of Ignatov et al., enriched in micro-metastases, an additional adjuvant treatment showed an effect on outcome reaching the baseline of the pN0-subgroup [24]. Unfortunately, isolated tumor cells were not studied in parallel.

The intense discussion of ultra-staging should question the role of pan-cytokeratin immunohistochemistry according to clinical relevance [10, 21]. Some meta-analyses showed an adverse prognostic effect of “low-volume” disease in SLN. However, they lumped together micro-metastases and isolated tumor cells and did not stratify for histological subtypes. Tumor-biologically, isolated tumor cells appear predominantly at the peripheral rim of the lymph node and were stronger associated with endometrioid subtype, low T-stage, and low grading. Lymph nodes are not yet enlarged and no histological proof of invasive capacity in terms of metastasis formation, desmoplasia, or distorted lymph node architecture could be observed. We found isolated tumor cells in high-grade serous carcinoma as well, but could not control for different biological behavior in different subtypes due to low case numbers. In theory, haphazard apoptotic tumor cell displacements could be hypothesized as well as single dormant cells with full metastatic potential. In total, our data with predominantly endometrioid cases with pN0(i+) cases showed no worsened survival so far.

### Limitations of the study — missing molecular data

In 2019, the WHO classification officially introduced molecular subgroups of POLE mutated, MMR deficient, and p53 aberrant, with the largest group being the non-mutational specific subgroup [34]. Due to this latest development, POLE mutational status was not regularly available, which represents the strongest limitation of our cohort. As a consequence, the applied ESGO risk classification of this study relies on the former definition without molecular data, which has been recently updated and put side to side for clinical decision making [11]. Of note, the addition of molecular analysis resulted in up- and down-staged ESGO risk classes of 2.9% and 3.7%, respectively [26]. Hence, the risk stratification presented here would not change in the majority, but could be sharpened.

Combined studies of molecular and intensified TNM classification investigation including SLN mapping are warranted, as precision in both fields will yield the greatest prognostic power.

Another limitation of this study is the lack of detailed information about adjuvant treatment and checkpoint inhibition in later cases. Regularly, our center did not change adjuvant treatment neither based on isolated tumor cells nor so far on micro-metastasis.

### Take home message for pathologists processing SLNs in endometrial carcinoma

SLN mapping has evolved as the method of choice to receive minimal nodal staging information in EC, whereas a therapeutic effect is still under investigation [7, 36, 39]. Nowadays, more than 5000 patients have been reported to be treated with this innovative surgical technique [6, 21]. The surgical approach merges indocyanine green and cervical injection site as the preferential operative method [27, 40]. Less than three SLNs per side has been proposed as an indicator for surgical experience — as it underlines straight-forward sampling before tracers are widely distributed in the tissue [36], but also pathologists tend to reduce workload in terms of less intensified work-up, if tracer diffusion is extensive. Pathology reports should outline the numbers of sentinel and regularly dissected lymph nodes separately, to provide these quality data to the surgical colleagues.

False negative SLNs exist rarely for downstream lymph nodes in the same sided region. However, contra-lateral and particular para-aortic lymph node involvement has been observed in approximately 5% of cases with negative SLN. This accounts for our cohort as well and has in parts already been investigated in surgical studies from our center [8, 25, 37]. From an anatomical point of view, a well-functioning SLN concept in the pelvic region can be assumed, but with a hard to reach privileged abdominal region comparable to the internal mammary lymph nodes in breast carcinoma.

Pathological processing should ensure the detection of any macro-metastases first. Our data support a gross perpendicular lamellation technique with slim slices of 2 mm to the best. Frozen sections of the SLN can be safely selected for particular case management and study context.

The next priority is micro-metastases, where evidence for prognostics and possible therapeutic implications was shown [24]. Protocols with wide-spaced levels, i.e., 200 μm, should be logically preferred. However, three levels with an optional pan-cytokeratin stain seem to be sufficient to achieve substantial detection rates [15].

So far, there are no prognostic or therapeutic consequences for isolated tumor cells [18, 21], which questions the role of pan-cytokeratin staining outside of prospective trials.

## Supplementary information

ESM 1 (DOCX 13 kb)

Supplemental Fig. 1 Frozen section (A) of lymph node metastasis in endometrial carcinoma with corresponding definite histology (B). The cohesive nature of tumor spread metastasis detection was safe and obvious during frozen section. (PNG 3038 kb)

High resolution image (TIF 4983 kb)

Supplemental Fig. 2 Different kind of pitfalls during ultrastaging of gynecological SLNs. Involvement of endometriosis or endosalpingiosis (A-D) with positivity for pancytokeratin, low proliferative growth and PAX8 positivity. Note the circular organized stromal reaction not resembling desmoplasia or infiltrative growth. Mesothelial proliferations (E-H) as contamination due to close peritoneum in SLN preparation. Negativity for BEREP4 in contrast to pancytokeratin and evidence for mesothelial markers, e.g. WT1. Cross-reactivity of primary or secondary antibodies with lymphocytes and plasma cells in scattered cells (I, K). (PNG 2469 kb)

High resolution image (TIF 3829 kb)
