# Supplementary material for: Pathological processing of sentinel lymph nodes in endometrial carcinoma — routine aspects of grossing, ultra-staging, and surgico-pathological parameters in a series of 833 lymph nodes
Source: Virchows Arch. 2022 Jul 19;481(3):421–32. doi: 10.1007/s00428-022-03377-6 (PMC9485184; doi:10.1007/s00428-022-03377-6)
Supplement: Supplementary file 1 — (DOCX 13 kb) [file 428_2022_3377_MOESM1_ESM.docx]

Supplemental information:

### **Histopathological pitfalls during evaluation for low-volume lymph node involvement**

Applying pan-cytokeratin staining to gyneco-oncological SLNs bears a risk of misinterpretation. Endometriosis and endosalpingiosis can be falsely assessed as positive lymph nodes. The presence of endometrial stromal cells, ciliated epithelium, lack of nuclear atypia and low proliferation could avoid an over-diagnosis. The close relation to the serosa surface during surgery could lead to attachments and possible inclusions of mesothelial cells affecting the lymph node, which will stain for pan-cytokeratin as well. Negativity of BerEp4 as well as positive stains for some mesothelial markers like Calretinin, D2-40, WT1 or MTAP might be warranted. Wrong impressions of isolated tumor cells could be explained by either post-processional skin sheds from the water bath or the cross-reactivity of primary and secondary antibodies, which could stain single lymphocytes, plasma cells or histiocytes. Some examples of our series are shown in Supplemental Figure 2.

**Additional immunohistochemistry**

Additional selective routine immunohistochemistry was applied for distinct differential diagnosis and pitfalls: BerEp4 (1:400, M0804 Dako, Santa Clara, CA, USA), PAX8 (1:800, 10336-1-AP, Proteintech, Rosemont, IL, USA), WT1 (1:400, 348M96 Cell Marque, Rocklin, CA, USA) and Ki67 (1:100, SP6, Cell Marque, Rocklin, CA, USA).

**Supplemental Figure 1:** Frozen section (A) of lymph node metastasis in endometrial carcinoma with corresponding definite histology (B). The cohesive nature of tumor spread metastasis detection was safe and obvious during frozen section.

**Supplemental Figure 2:** Different kind of pitfalls during ultrastaging of gynecological SLNs. Involvement of endometriosis or endosalpingiosis (A-D) with positivity for pancytokeratin, low proliferative growth and PAX8 positivity. Note the circular organized stromal reaction not resembling desmoplasia or infiltrative growth. Mesothelial proliferations (E-H) as contamination due to close peritoneum in SLN preparation. Negativity for BEREP4 in contrast to pancytokeratin and evidence for mesothelial markers, e.g. WT1. Cross-reactivity of primary or secondary antibodies with lymphocytes and plasma cells in scattered cells (I, K).
