# Supplementary figures and images for: Pathological processing of sentinel lymph nodes in endometrial carcinoma — routine aspects of grossing, ultra-staging, and surgico-pathological parameters in a series of 833 lymph nodes
Source: Virchows Arch. 2022 Jul 19;481(3):421–32. doi: 10.1007/s00428-022-03377-6 (PMC9485184; doi:10.1007/s00428-022-03377-6)

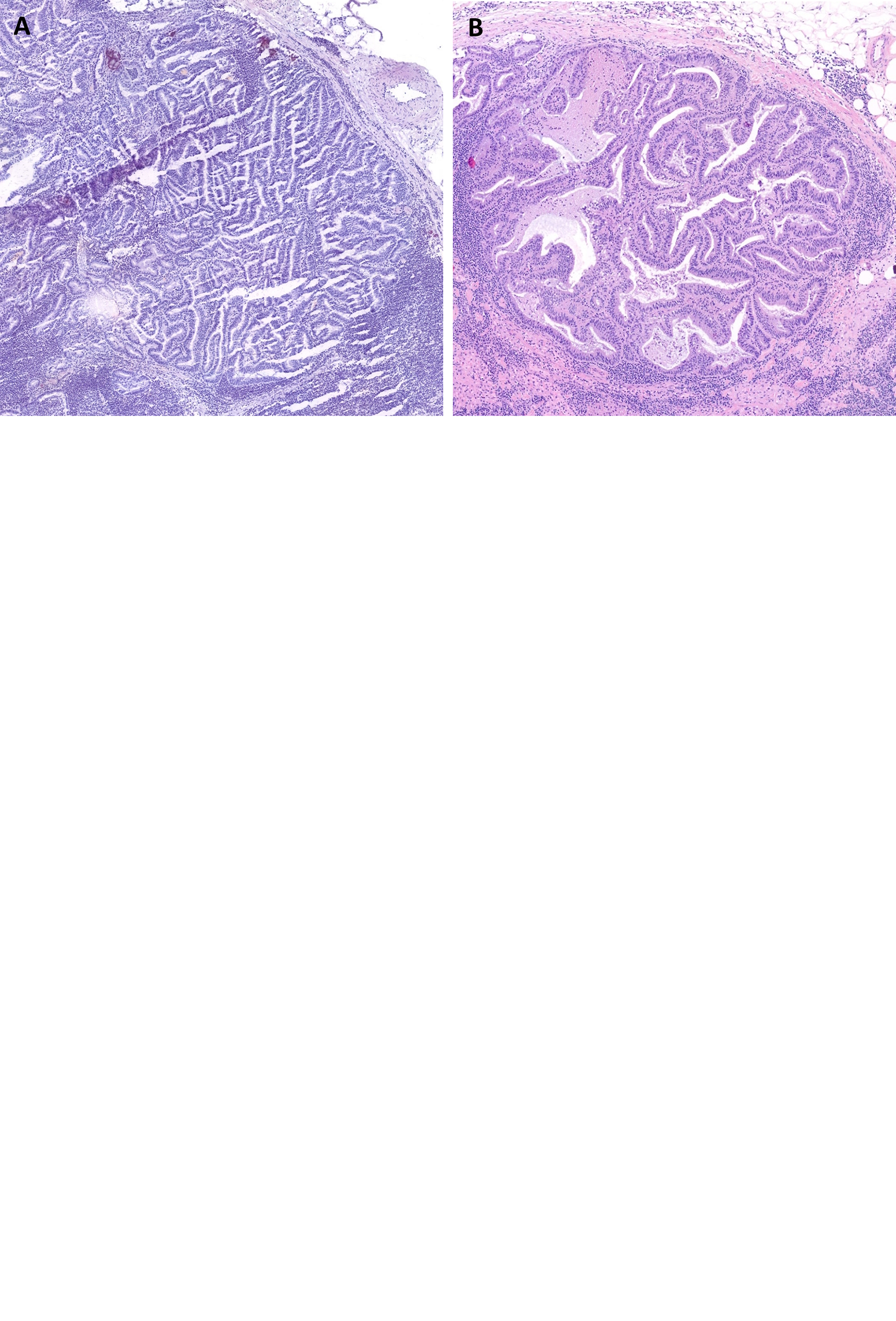

Supplement: Supplementary file 2 — Frozen section (A) of lymph node metastasis in endometrial carcinoma with corresponding definite histology (B). The cohesive nature of tumor spread metastasis detection was safe and obvious during frozen section. (PNG 3038 kb) [file 428_2022_3377_Fig6_ESM.png]

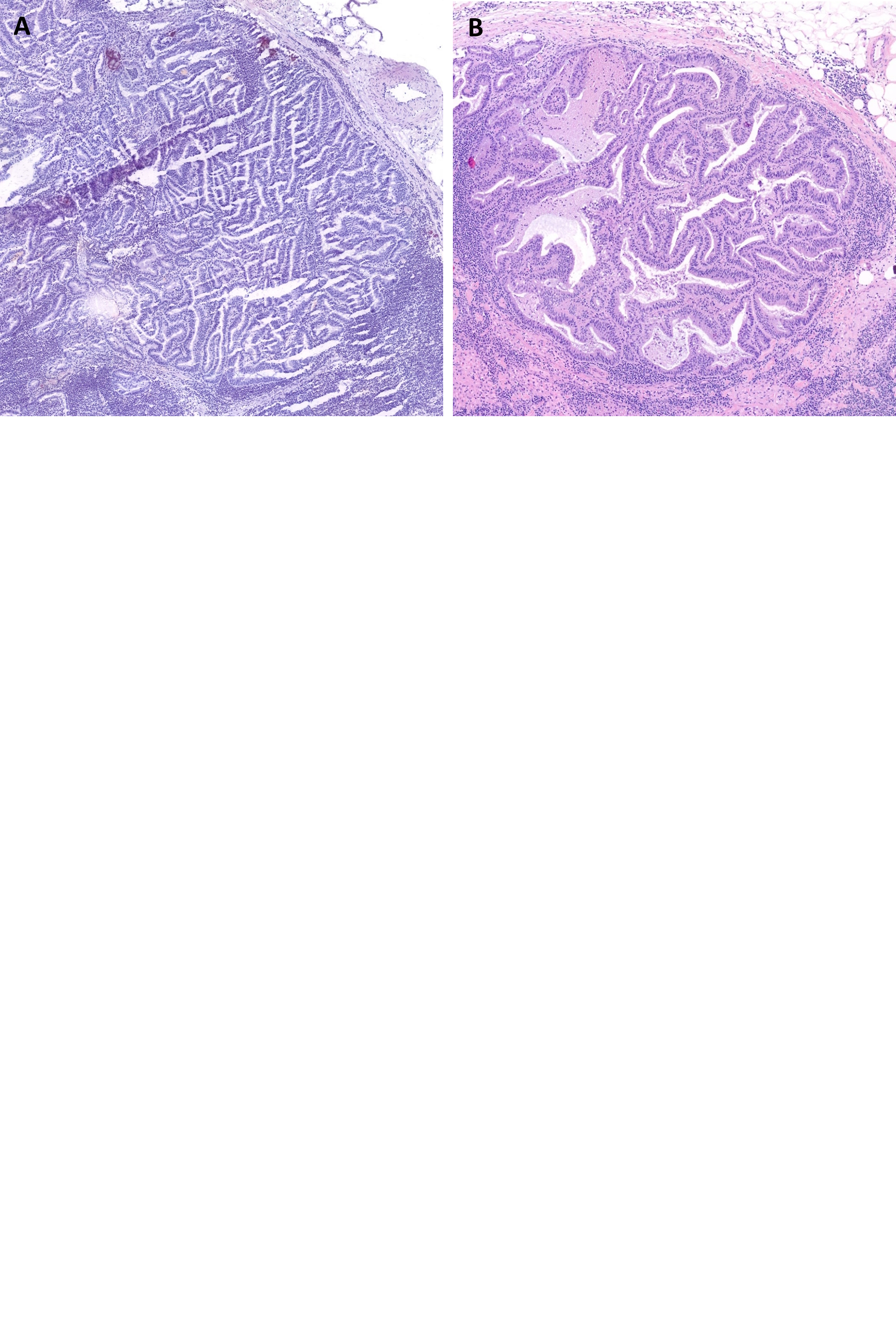

Supplement: Supplementary file 3 — High resolution image (TIF 4983 kb) [file 428_2022_3377_MOESM2_ESM.tif]

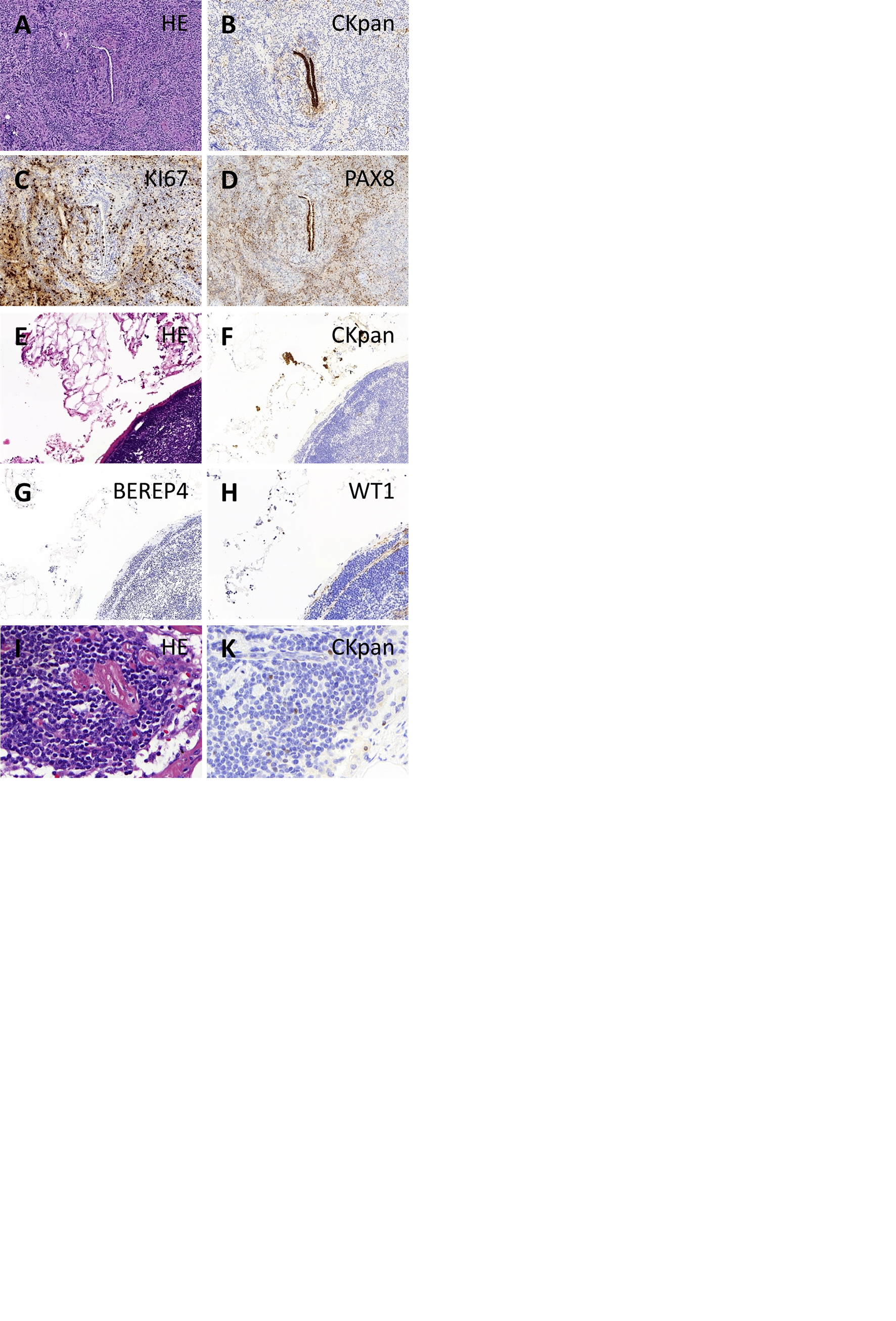

Supplement: Supplementary file 4 — Different kind of pitfalls during ultrastaging of gynecological SLNs. Involvement of endometriosis or endosalpingiosis (A-D) with positivity for pancytokeratin, low proliferative growth and PAX8 positivity. Note the circular organized stromal reaction not resembling desmoplasia or infiltrative growth. Mesothelial proliferations (E-H) as contamination due to close peritoneum in SLN preparation. Negativity for BEREP4 in contrast to pancytokeratin and evidence for mesothelial markers, e.g. WT1. Cross-reactivity of primary or secondary antibodies with lymphocytes and plasma cells in scattered cells (I, K). (PNG 2469 kb) [file 428_2022_3377_Fig7_ESM.png]

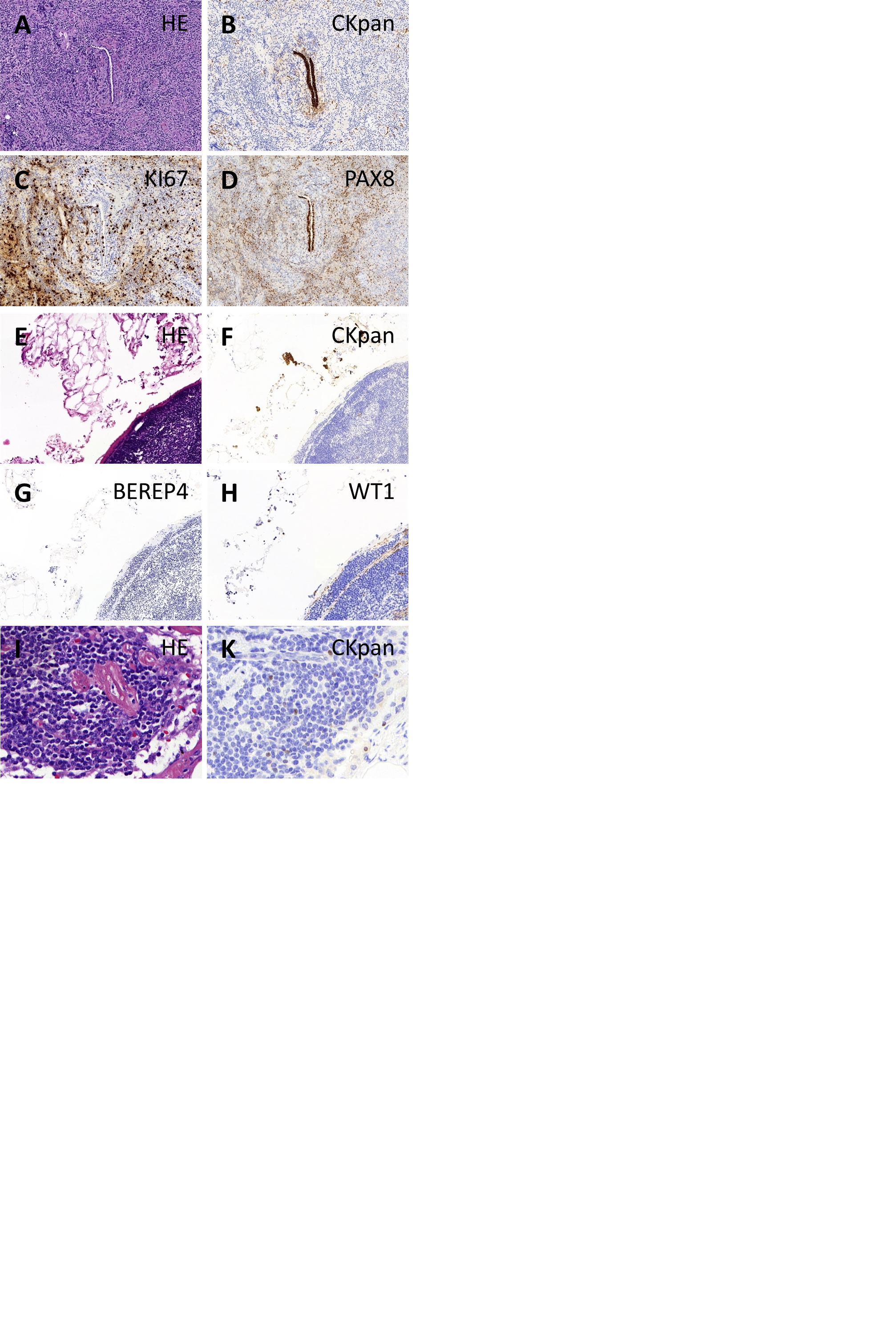

Supplement: Supplementary file 5 — High resolution image (TIF 3829 kb) [file 428_2022_3377_MOESM3_ESM.tif]
